# MIDClass: Microarray Data Classification by Association Rules and Gene Expression Intervals

**DOI:** 10.1371/journal.pone.0069873

**Published:** 2013-08-06

**Authors:** Rosalba Giugno, Alfredo Pulvirenti, Luciano Cascione, Giuseppe Pigola, Alfredo Ferro

**Affiliations:** 1 Department of Clinical and Molecular Biomedicine, University of Catania, Catania, Italy; 2 Department of Molecular Virology, Immunology and Medical Genetics, Comprehensive Cancer Center, The Ohio State University, Columbus, Ohio, United States of America; Indiana University, United States of America

## Abstract

We present a new classification method for expression profiling data, called MIDClass (Microarray Interval Discriminant CLASSifier), based on association rules. It classifies expressions profiles exploiting the idea that the transcript expression intervals better discriminate subtypes in the same class. A wide experimental analysis shows the effectiveness of MIDClass compared to the most prominent classification approaches.

## Introduction

Microarrays are a well established technology to analyze the expression of many genes in a single reaction whose applications range from cancer diagnosis to drug response. They are matrices, where known samples of DNA, cDNA, or oligonucleotides, called probes, combine with mRNA sequences. The expression level of genes is given by the amount of mRNA bounding to each entry. The aim is to find either sets of genes that characterize particular disease states or experimental condition or highly correlated genes that share common biological features. Microarray numerical data coming out from experiments are normalized and analysed [1]. Several algorithms and methods have been used for this analysis [2]. In particular statistical models should be suitable to correctly estimate the magnitude and the significance of differentially expressed genes [3]–[5]. Finally, specific supervised and unsupervised learning methods allow to investigate the predictive power of the candidate gene sets [6]–[8]. Several successful classification methods have been reported in the literature [9]–[13].

Support Vector Machines (SVM) [9] are powerful binary classification methods which take as input a training set of data, each belonging to one of two given classes. It finds support vectors for the classification by identifying a maximum separating hyperplane either when data are linearly separable or through kernel functions. SVMs can be successfully applied to multicategorial classification by using the “one-against-all” methodology [14].

Decision trees [10] are hierarchical models for supervised learning based on the idea that classification can be broken down into a set of progressive choices on the attributes. In a decision tree each internal node denotes a test on an attribute, each branch gives the outcome of the test and each leaf node stores the class label. Each path from the root to a leaf corresponds to a classification decision. When single tree classification shows low predicting power then decision forest classification can improve accuracy. Random Forests (RF) [13] build hundreds of trees. Each tree refers to a random variant of the same data. A single tree in the forest is built by using a bootstrap sample obtained from the training set.

The Nearest-Neighbor classifier [15] assigns to an unknown phenotype the label associated to the nearest sample tuple. The natural extension of the nearest-neighbor rule is the 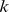-Nearest-Neighbor classifier (k-NN). In this case, the new tuple label will be the most represented in the 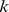-nearest-neighbor tuples. The distance from the new tuple is used as a weight in the classification. This method tends to be slow for large training sets.

Diagonal Linear Discriminant Analysis [16] (DLDA) is a linear discriminant analysis method with future selection based on a diagonal covariance matrix which ignores potential correlation between different features.

Those classifiers make use of a so called black-box and rely on many genes to give good classification results. However, recently, Wang and Simom [17] explored the virtues of very simple single gene classification models for molecular classification of cancer. They first identify the genes with the most powerful univariate class discrimination ability and then construct simple classification rules for class prediction using those single genes. Their results show that in many cases the single gene classification yields more accurate classification results than classical approaches.

In this paper we present a new classification method, called MIDClass (Microarray Interval Discriminant CLASSifier) based on association rules. Association Rule Mining has been proven to be effective in many microarray applications[18]–[20]. In [18], authors extract significant relations among microarray genes annotated with metabolic pathways, transcriptional regulators and Gene Ontologies. In [19], McIntosh and Chawla employ quantitative association rules capable of dealing with numeric data representing cumulative effects of variables. In [20], Antonie and Bessonov classify using feature selection based on Support Vector Machines with recursive feature elimination in connection to association rules.

**Figure 1:**
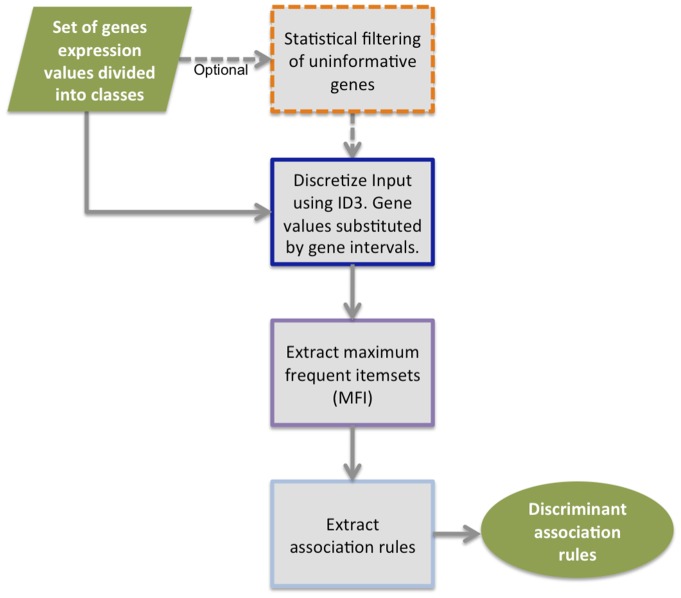
MIDClass flowchart.

MIDClass is an association rule mining method [18], [19], [21] which classifies gene expression exploiting the idea that *gene expression interval values could better discriminate subtypes in the same class*. A flowchart of the proposed method is pictured in Figure 1. A wide experimental analysis shows the effectiveness of such a method compared to the above most prominent classification approaches. MIDClass is available at http://ferrolab.dmi.unict.it/midclass.html.

## Methods

### Overview

Association rule mining finds sets of items (called frequent itemsets) whose occurrences exceed a predefined threshold in the dataset. Then it generates association rules from those itemsets with the constraints of minimal confidence. The *market basket analysis* [22] problem is an example of this kind of mining. Customers habits are classified by finding associations between the items placed in their shopping baskets.

In MIDClass items are gene expression intervals. Baskets are the phenotypes containing (i.e. described by) sets of gene expression intervals. The aim of MIDClass is to extract frequent maximal itemsets and then use them as rules whose antecedent is the conjunction of gene expression intervals and the consequence is the class-label.

**Figure 2:**
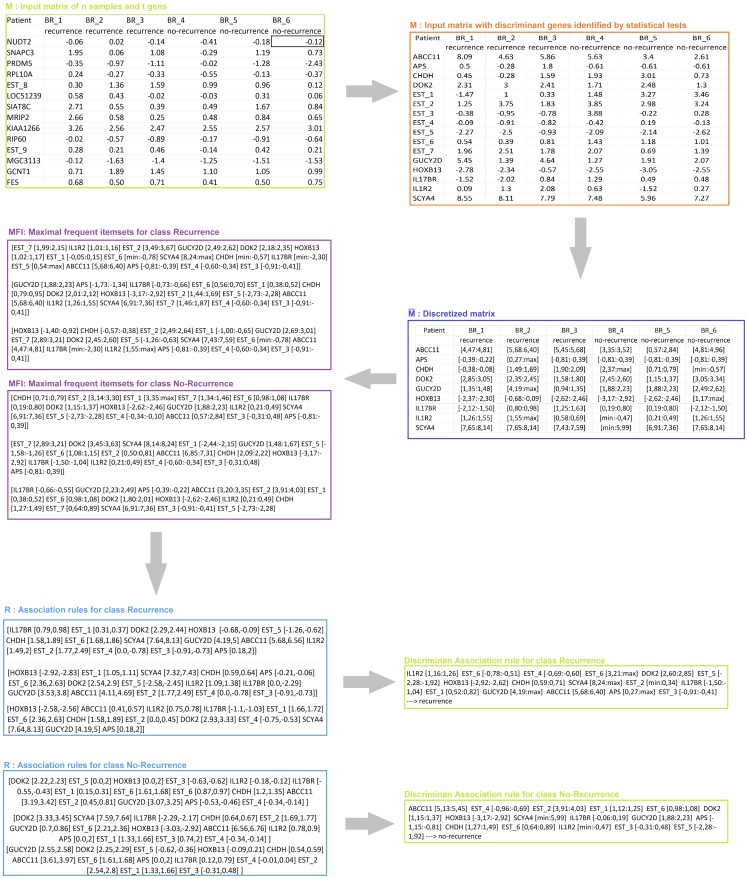
Example of MIDClass flowchart on Breast Cancer 2 Dataset (data are partially shown). Let 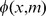 denote the expression value of sample 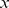 on the 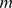-th gene (an example of entry in M is shown as a black box). Samples are divided into classes corresponding to phenotypes disease. After discretization process, MIDClass constructs a matrix 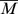 from 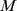 by replacing each 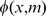 with the unique interval containing it. 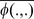 denotes an entry in 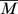 (an example of entry in 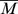 is shown as a black box). Then, MIDClass computes per class the possible sets of 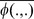 that are frequent and they have maximal size. MIDClass filters out gene expression intervals which size are below a given threshold. Since, association rules express interesting relationships between gene expressions and class labels, MIDClass uses them for classification. Therefore, MIDClass extracts a set of rules per class. Each rule has quantitative attributes on the antecedence part (i.e. discretized values) and one categorical attribute on the consequence side (i.e. the class 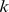). Finally, it returns only rules that have a maximal score. The score takes into account the number of items in each sample are contained in the rule together with the cardinality of the rule (the computation of the score is described in detailed in the Methods section).

Before mining, MIDClass preprocesses data. First, MIDClass applies the T-Test [23], [24] to filter those genes whose expression do not present any significant variability across the classes. Next, a discretization algorithm partitions the gene expression intervals into subintervals possessing strong discriminant power in each class. A flowchart of the proposed method on “Breast Cancer 2” Dataset is depicted in Figure 2.

### Data format

MIDClass takes as input a matrix 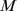 of 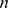 samples 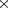 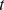 genes. Let 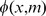 denote the expression value of sample 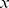 on the 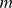-th gene, with 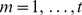. Samples are divided into classes corresponding to phenotypes disease. Let 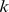 be a class label, 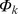 is the set of all values 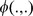 of genes associated to phenotypes of class 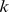.

### Statistical gene filtering

In order to select discriminant gene expression values MIDClass applies the T-Test [23], [24]. This step is critical for the reliability of the results, since the presence of not informative genes might negatively affect the classification and the computational performances. Notice that this step is done before running MIDClass . Other suitable statistical test can be applied. In particular, MIDClass uses the LIMMA package available in R through Bioconductor [25].

### Discretization

For each gene 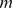, MIDClass partitions its expression value 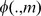 by a discretization algorithm. This produces a set of intervals and each expression value belongs to only one of them. Consequently, MIDClass constructs a matrix 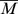 from 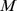 by replacing each 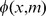 with the unique interval containing it.

To perform such a discretization, since there is no best discretization method, MIDClass includes the following techniques [26]: ID3, EWIB, NONE. However, among the available discretization algorithms, in the tested datasets, ID3 showed to be one of the more robust. MIDClass uses ID3 as default discretization algorithm.

We denote by 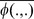 the entries in 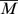, with 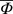 the set all possible 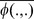 in 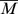, and with 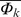 the set of intervals falling into the 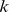-th class. Note that, MIDClass classifies 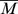, i.e. it mines gene expression intervals rather than gene expression values.

### Extract maximal frequent itemsets

Let 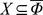 be an itemset (a set of pairs composed by gene and expression interval) and let 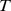 be a collection of itemsets (e.g. the set all possible itemsets). We denote by 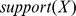 the percentage of itemsets 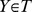 such that 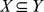. The support measures how often 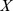 occurs in 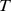. Let 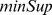 denote a threshold value given by the user whose experimentally default value is 0.4. If 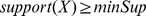, then 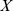 is claimed as a frequent itemset.

Let 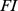 be the set of all frequent itemsets in 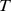. If 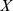 is frequent and there is no frequent superset of 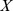, then 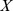 is a *maximally frequent itemset*. We denote by 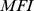 the set of all maximally frequent itemsets. MIDClass extracts only 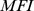
s by using the MAximal Frequent Itemset Algorithm (MAFIA) [27].

Since gene expression intervals may be too narrow and some time with a low significance, MIDClass allows users to tune the model by filtering out those that are below a certain threshold (Minimal Interval Size threshold – the default value is 0.05).

### Extract association rules from maximal frequent itemsets

An association rule is an implication 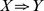, where 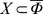, 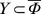, and 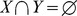. Let 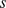 be the percentage of subsets in 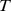 containing 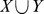 and let 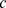 be the percentage of subsets of 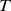 containing both 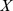 and 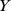. Then, 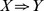 holds in the set 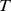 with support 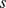 and it has confidence 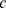 in 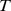. The minimum confidence is a threshold value given as input by the user and whose default value is 0.05.

Association rules express interesting relationships between gene expressions and class labels. Therefore, MIDClass uses them for classification.

MIDClass identifies maximal frequent itemsets for each class 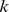, 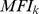, by using 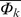. Those itemsets generate the set of rules 
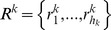
 per class. The antecedence of each rule is the conjunction of the items (gene and expression interval) and the consequence is the membership of class 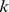.

### Extract discriminant association rules and classification

Let 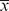 be an unknown discretized sample (i.e. genes expressions are represented by intervals), and let 
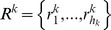
 be the association rules for class 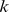. For 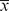, MIDClass evaluates how many rules are satisfied, even partially, in each 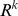.

MIDClass assigns 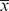 to that class 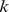 whose rules are *maximally satisfied* by the following scoring function.

Given a class 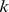, MIDClass first evaluates 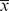 for each rule 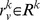, by using the following function 
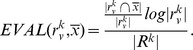
 Notice that, 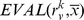 takes into account the number of items in the sample contained in the antecedent of the rule together with the cardinality of the rule 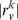. The value is normalized by the number of rules, 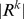, in the class 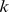. Finally, the score assigned to the sample 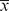 with respect to the class 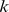 is set to be 
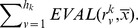


## Results and Discussion

All tests have been run on a HP Pavilion with Intel Corei7, 8GB RAM and ubuntu 12.04.

### Datasets and Preprocessing

We selected eleven gene expression datasets used in [17]. All datasets are publicly available and were downloaded from the BRB-Array Tools Data Archive for Human Cancer Gene Expression repository 2 (http://linus.nci.nih.gov/brb/DataArchive_New.html). In Table 1 we give the details of each dataset. In Table 2 we report the number of genes used by each classifier. Finally, in oder to compare MIDClass with the association rule mining based method reported in [20] we used the same Leukemia dataset [28]. Leukemia dataset contains the gene expression profile from the leukemia microarray study of Golub et al. [28]. It consists of 72 bone marrow tissues: 47 acute lymphoblastic leukemia (ALL) cases and 25 acute myeloid leukemia (AML) cases.

**Table 1:** Dataset description.

Dataset	Description
Brain Cancer	60 samples, 46 patients with classic and 14 patients with desmoplastic brain cancer
Breast Cancer 1	99 samples, patients that did (n = 45) and did not relapse (n = 54)
Breast Cancer 2	60 samples, disease-free (n = 32) or cancer recurred (n = 38)
Gastric Tumor	132 samples, 103 tumor samples and 20 normal controls
Lymphoma	58 samples. Patients that did (n = 32) and did not cured (n = 26)
Lung Cancer 1	41 samples, squamous cell lung carcinoma (21) or pulmonary carcinoid (20)
Lung Cancer 2	181 samples, 31 mesothelioma samples and 150 adenocarcinoma
Melanoma	70 samples, 45 cases of malignant melanoma patients and 25 of non-malignant patients
Myeloma	173 samples, 137 patients with bone lytic lesions,36 patients without
Pancreatic Cancer	49 samples, 24 ductal carcinoma samples and 24 normal controls
Prostate	Cancer 102 samples, 50 non-tumor prostate and 52 prostate tumors

**Table 2:** Number of genes used by classifiers in each tested dataset.

Dataset	MIDClass	SGC-t	SGC-W	DLDA	k-NN	SVM	RF
Melanoma	55	1	1	7200	7200	7200	7200
Breast Cancer 1	8	1	1	17	17	17	15
Brain Cancer	239	1	1	14	14	14	14
Breast Cancer 2	16	1	1	176	176	176	176
Gastric Tumor	23	1	1	848	848	848	848
Lung Cancer 1	101	1	1	7472	7472	7472	7472
Lung Cancer 2	55	1	1	3207	3207	3207	3207
Lymphoma	3	1	1	2	2	2	2
Myeloma	27	1	1	169	169	169	169
Pancreatic Cancer	22	1	1	56	56	56	44
Prostate Cancer	45	1	1	798	798	798	798

### Performances

After MIDClass had generated the rules, we filter each rule by removing the genes intervals whose presence did not give any classification improvement. This step is implemented by applying a Leave-One-Out gene strategy [29].

To motivate the usage of gene intervals we run MIDClass on genes values discretized by using 1 for up regulated genes, −1 for donwregulated and 0 for normal expression values. We tested this model on the Breast Cancer 2 dataset obtaining very poor results (46% of accuracy).

**Figure 3:**
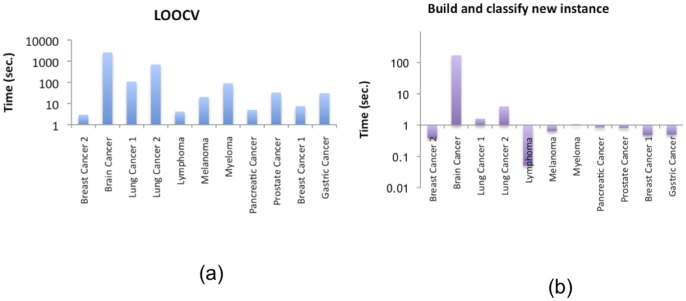
Runninig time of MIDClass to (a) build and establish its reliability using the LOOCV and (b) to create the model and classify a new instance.

Concerning MIDClass running time, Figure 3 (a) reports the running time to build and establish the reliability of the model using the LOOCV on the tested dataset and Figure 3 (b) the time to execute MIDClass to create the model and classify a new instance.

**Figure 4:**
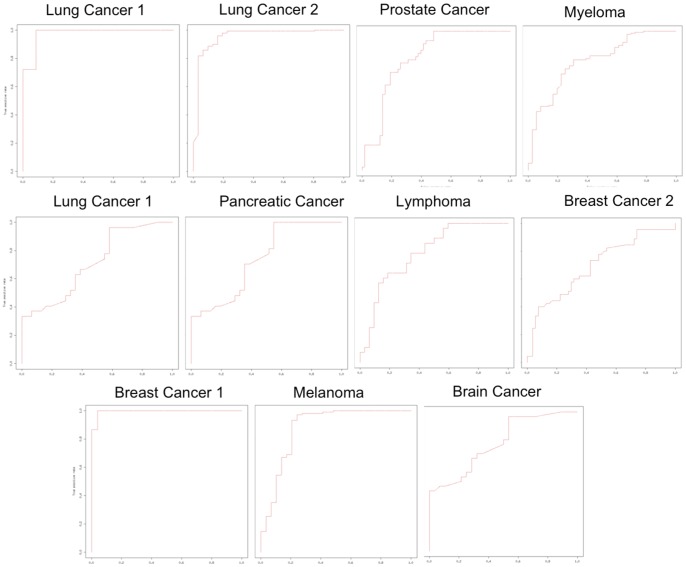
MIDClass ROC curves.

We compared our system against competitors using a Leave-One-Out-Cross-Validation (LOOCV). Using cross-validation one can better assess the performance of the classifier and predict how the classifier will generalize to a new independent data set. Table 3 reports the average accuracy obtained from LOOCV. Figure 4 reports the ROC curve of MIDClass on all the analyzed datasets.

**Table 3:** Comparisons of MIDClass , single gene classifiers and standard classifiers.

Dataset	MIDClass	SGC-t	SGC-W	DLDA	k-NN	SVM	RF
Melanoma	**98.5** (ID3, 0.1, 2)	97	96	97	97	97	97
Breast Cancer 1	**76** (ID3, 0.05, 1)	63	69	61	53	52	43
Brain Cancer	**83** (ID3, 0.01, 1)	80	77	65	73	60	70
Breast Cancer 2	**90** (ID3, 0.05, 1)	58	50	73	67	73	67
Gastric Tumor	94 (ID3, 0.05, 2)	89	80	81	96	**97**	95
Lung Cancer 1	**98** (ID3, 0.05, 2)	**98**	95	95	**98**	**98**	**98**
Lung Cancer 2	**99** (ID3, 0.01, 2)	93	93	**99**	**99**	**99**	**99**
Lymphoma	69 (ID3, 0.1, 2)	**76**	71	66	52	59	57
Myeloma	**84** (ID3, 0.05, 2)	68	67	75	78	74	79
Pancreatic Cancer	78 (ID3, 0.05, 1)	69	**90**	63	61	65	55
Prostate Cancer	92 (EWIB, 0.01, 2)	89	89	78	**93**	**93**	**93**

We report the average accuracy of all tested classifiers on the selected dataset obtained with standard LOOCV. The performances concerning the compared algorithms have been retrieved from [17]. Concerning MIDClass , in brackets we report the discretization algorithm, the MFI threshold and the 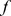 function (1: 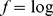 2: 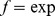).

Wang and Simon in [17] (here after Single Gene Classify) claim that in most of the cases a single gene is enough to obtain a better classification compared to the state of the art. However, we show that MIDClass outperforms the Single Gene Classify ( based on t-test (SGC-t) and based on WMW (SGC-W)) in almost all cases and in particular on those datasets in which it had poor performances (see Table 3). Table 3 shows also that MIDClass outperformed the standard methods (DLDA, k-NN, SVM and RF). The performances of compared systems have been obtained from [17]. Results in Table 3 also show that MIDClass in connection with ID3 in all datasets but one, Prostate Cancer, is more performant than EWIB. Finally, MIDClass in almost all cases outperformed all compared systems.

Comparing with the method in [20] in the Leukemia dataset, we observed that our rules have a high number of genes (an average of 10 genes compared to the 5 reported by the authors) and some genes were not present in our rules (such as RIN2,MNX1,LYN,GSTT2, GJA5,CD63 and CYFIP2). However, MIDClass yields a better classification performance ( MIDClass : 97.2% vs method in [20]: 95.52%).

Although MIDClass has been designed to afford two-class classification problems, we tested its performances in a multi-class classification problem. Following [14] we implemented the algorithm according to the One-Versus-All strategy. We performed a LOOCV on the SRBT dataset [30] obtaining 100% classification accuracy by using ID3, MFI threshold equals to 0.05 and Minimal Interval Size equals to 0.05. By comparing such results with the one yielded by ANMM4CBR [31] (around the 97%), MIDClass looks promising also for classifying multi-class instances.

Finally, in order to validate the use of gene intervals we conducted an experiment by substituting the intervals assigned to genes in the rules with their fold changes. We observed a strong degradation of the performances. Table 4 presents some of the MIDClass classification rules that have been used in the classification process of “Breast Cancer 2” dataset. For example, by substituting the intervals assigned to APS and IL17BR with their fold change we obtained a poorer classification performance equal to 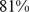 (originally it was 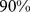).

**Table 4:** MIDClass classification rules in breast cancer 2 dataset.

Rule	Genes	Class
Rule1	IL17BR[0.79,0.98], DOK2[2.29,2.44], HOXB13[−0.68,−0.09], CHDH[1.58,1.89],	
	SCYA4[7.64,8.13], GUCY2D[4.19,2.14E7], ABCC11[5.68,6.56], IL1R2[1.49,2.14E7],	
	APS[0.18,2.14E7]	NonRecurrence
Rule2	ABCC11[2.84,3.19], IL17BR[0.0,2.14E7], CHDH[0.94,1.2], GUCY2D[3.53,3.8],	
	SCYA4[7.64,8.13], APS[0.18,2.14E7]	NonRecurrence
Rule3	DOK2[2.23,2.25], APS[−0.46,−0.38], IL1R2[1.09,1.38], IL17BR[0.0,−2.29],	
	SCYA4[8.16,2.14E7], ABCC11 [5.68,6.56], HOXB13[1.1,2.14E7]	NonRecurrence
Rule4	IL17BR[−0.43,−0.34], CHDH [0.0,2.14E7], SCYA4[6.91,7.06], APS[−0.74,−0.64],	
	GUCY2D[4.19,2.14E7], HOXB13[1.1,2.14E7]	NonRecurrence
Rule5	GUCY2D[0.56,0.7], APS[−1.34,−1.15], HOXB13[−2.2,−2.09], DOK2[2.0,2.11],	
	ABCC11[4.96,5.25], SCYA4[6.91,7.06], CHDH[1.58,1.89]	NonRecurrence
Rule6	HOXB13 [−0.09,0.21], ABCC11 [3.61,3.97],APS [0.0,2.14E7],IL17BR [0.12,0.79]	Recurrence
Rule7	GUCY2D [2.75,2.84],HOXB13 [0.56,0.85],ABCC11 [3.44,3.51], IL17BR [−1.03,−0.76],	
	CHDH [1.2,1.35],APS [0.0,2.14E7],DOK2 [1.21,1.45]	Recurrence
Rule8	IL17BR [1.18,1.24],ABCC11 [0.0,2.14E7],APS [0.0,2.14E7], GUCY2D [3.07,3.25],	
	DOK2 [0.0,1.2],CHDH [1.89,2.15],HOXB13 [−2.77,−2.58], IL1R2 [0.0,−0.37]	Recurrence
Rule9	GUCY2D [2.0,2.41], IL17BR [1.46,2.14E7],APS [−0.53, −0.46],CHDH [2.36,2.14E7],	
	ABCC11 [0.57,2.84]	Recurrence
Rule10	SCYA4 [0.0,5.99], DOK2 [0.0,1.2],IL17BR [0.12,0.79] ,IL1R2 [0.0,−0.37],	
	APS [−1.15,−0.74]	Recurrence

### Discussion of Biological Relevance

Table 4 presents some of the classification rules that have been used in the classification process of breast cancer 2 dataset. We assessed each gene in the rules in the breast cancer context by reviewing relevant literature and using IPA-Ingenuity Software (http://www.ingenuity.com/).

Most part of genes in Table 4 were related to breast cancer. For example, HOXB13, IL17BR and CHDH genes represented by Rule1 are correlated with ER status and all three genes exhibited an ER-dependent correlation pattern with HER2 status.

ER is a member of the nuclear hormone family of intra cellular receptors, it is a DNA-binding transcription factor which regulates gene expression. Binding of estrogen to ER stimulates proliferation of mammary cells, producing an increasing of cell division, DNA replication, and increases mutation rate. This causes disruption of the cell cycle, apoptosis and DNA repair processes eventually leading to tumor formation. The Human Epidermal growth factor Receptor 2 HER2/neu belongs to a family of four trans membrane receptor tyrosine kinases involved in signal transduction pathways that regulate cell growth and proliferation. Over-expression of this receptor in breast cancer is associated with increased disease recurrence and worse prognosis.

Previous studies shown that HOXB13 is negatively regulated by ER, likely through a different mechanism than gene activation[32]. On the contrary, IL17BR and CHDH are both positively correlated with ER expression, their extent of correlation is less than that for PR and pS2. Previously, the HOXB13:IL17BR index was found to be higher in HER2-positive tumors than that in HER2-negative tumors [33]. The relationship of these three E2-regulated genes to HER2 is notable because recent data from preclinical models indicate that crosstalk between the HER2 and ER signaling pathways directly contributes to the development of tamoxifen resistance [34], [35]. They are estrogen-regulated genes, and have a prognostic utility due to their complex regulation through both ER- and HER2-dependent pathways. These two pathways play a key role in breast cancer.

Another relevant gene reported by Rule10 is CCL4 (Gene Symbol SCYA4). It is a member of chemokines family and was present at high levels in breast cancer tissues compared to normal tissues [36]. This gene with CC chemokines CCL2 is also correlated to the grade of breast tumors [37]. High levels of CCL2 or CCL4 trigger macrophage, B and T lymphocytes recruitment to the tumor [36], [38], which is correlated with a poor prognosis [36]. A previous study also showed that CCL2 was correlated to lymph node status [38]. CCL2 -2518A/G promoter polymorphism has been shown to be correlated with staging and metastasis of breast cancer patients [39]. CCL4 displayed an expression inversely correlated to ER and to PR in breast cancer biopsies and is linked to HER2 status.

ABCC11 is associated with resistance to methotrexate and fluoropyrimidines, two classes of agents widely used for breast cancer treatments (see Rule10). Its transcripts were overexpressed in estrogen receptor-(ER-) positive breast cancers [39]. ABCC11-mediated transport of anticancer drugs, combined with its expression levels in a hormonally-regulated breast tissue, suggest that the pump expression may be regulated by xenobiotics. ABCC11 mRNA and protein levels were enhanced by DEX (dexamethasone) a potent anti-inflammatory factor widely used in cancer therapy, and by PROG (progesterone) in MCF7 (progesterone receptor-(PR-) positive) but not in MDA-MB-231 (PR-negative) breast cancer cells. This suggested a PR-signaling pathway involvement in ABCC11 regulation. Furthermore, ABCC11 levels were positively correlated with the PR status of postmenopausal patient breast tumors from two independent cohorts. Thus, in the subclass of breast tumors (Estrogen Receptor-(ER-) negative/PR-positive), the elevated expression level of ABCC11 may alter the sensitivity to ABCC11 anticancer substrates, especially under treatment combinations with DEX [40]–[42].

Interesting interaction between DOK2 and APS is represented by Rule10. These genes are not yet related to breast cancer although they are missexpressed in several breast cancer mRNA profiling. DOK2 is known as the substrate of chmeric p210bcr/abl oncoprotein characterizing chronic myelogenous leukemia with Philadelphia chromosome. Reduced DOK2 expression was recently reported in lung adenocarcinoma, suggesting that this protein acts as a tumor suppressor in solid tumors.

Finally, we mark that the use of gene intervals instead of gene fold changes not only improves the classification power of MIDClass as shown in Performances Section but reflect also the two operating modes of gene expression at the messenger level: baseline, and under-expression or over-expression. In addition, converting real gene expression data into a typically small number of finite values maintaining the variation of the original data, generates more intuitive rules that are able to catch possible individual variation.

## Supporting Information

Manual S1 **MIDClass user manual.**
(PDF)
