# Supplementary material for: MIDClass: Microarray Data Classification by Association Rules and Gene Expression Intervals
Source: PLoS One. 2013 Aug 6;8(8):e69873. doi: 10.1371/journal.pone.0069873 (PMC3735555; doi:10.1371/journal.pone.0069873)
Supplement: Manual S1 — MIDClass user manual. (PDF) [file pone.0069873.s001.pdf]

How to install and use MIDClass

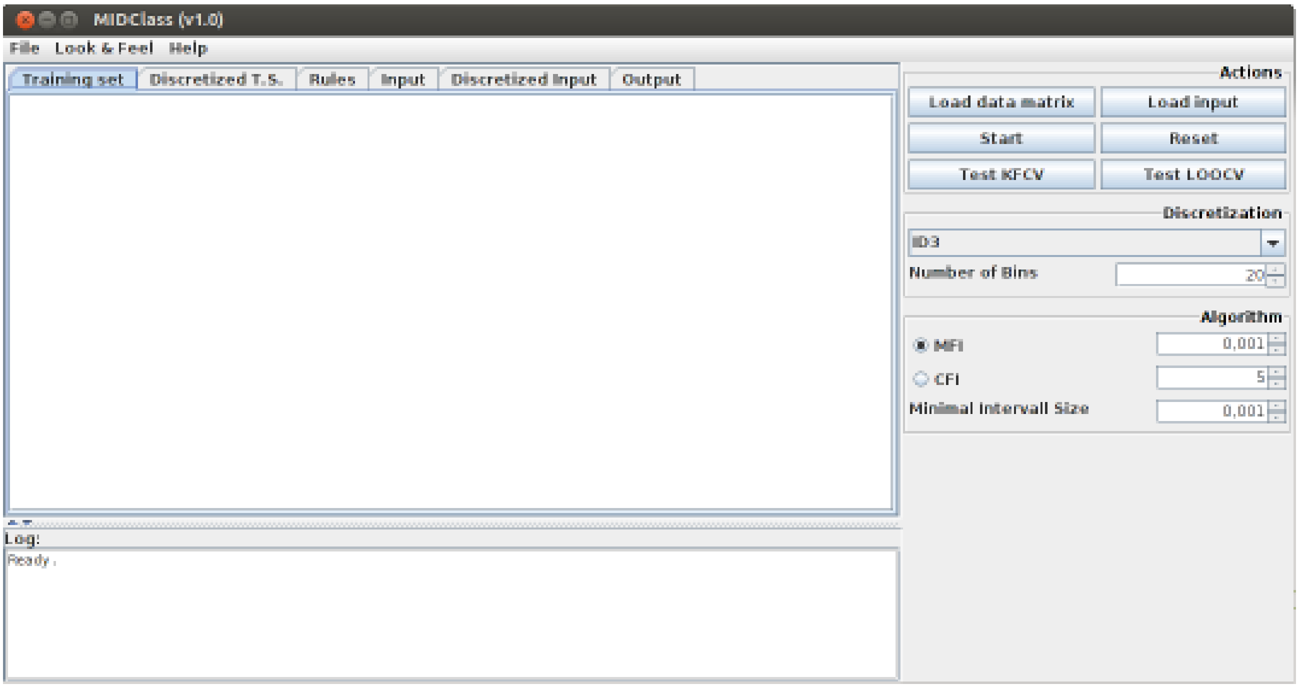

There are different ways to get MIDClass running:

- Installing locally and executing it
- Compiling the source code

Java Jar File

A **JAR** (**J**ava **AR**chive) is an archive file format typically used to aggregate many Java class files and associated metadata and resources (text, images and so on) into one file to distribute application software or libraries on the Java platform.

A JAR file allows Java runtimes to efficiently deploy a set of classes and their associated resources. The elements in a JAR file can be compressed, which, together with the ability to download an entire application in a single request, makes downloading a JAR file much more convenient than separately downloading the many uncompressed files which would form a single Java Application.

To lunch the program, users have to enter this command similar to "java -jar nameOfJarFile.jar".

Installing locally

You can download MIDClass from http://ferrolab.dmi.unict.it/midclass.html
You will get a zip file that can be installed and executed in any Linux operating systems.
The first thing to do is to uncompress the zip file into some folder. Use your preferred application for this, usually doing click or double click over it will start the application that will allow you to uncompress it.

Once uncompressed you will get a folder like MIDClass (the name of the folder could be different depending on the name chose).
That's all, you have already installed MIDClass in your computer and you can work with the graphical interface.

To run MIDClass graphical interface go inside its folder open a terminal and type:

- java -jar MIDClass.jar

Prerequisites

You need to have java installed. Go to this website to download and install; http://www.java.com/en/download/ . You need to add path of java after set up; go here to do this: http://www.java.com/en/download/help/path.xml. This will tell your computer where to find the java program. The current version of MIDClass are available for Linux/Unix OS and require the pre-installation of MAFIA 1.4. This package can be downloaded via: http://himalaya-tools.sourceforge.net/Mafia/
After downloading the tar.gz file, please extract it and put its content into the folder /MIDClass/fi/Mafia-1.4. Then, you need to compile Mafia package. To do that, please follow these steps:

1. Open a terminal and place into the folder fi/Mafia-1.4 present in to the MIDClass folder.
2. Type 'make' to compile the package
3. Type 'make install' to install the programs and any data files and documentation
4. Move the file mafia presents in MIDClass /fi/Mafia-1.4/src in MIDClass/fi

Memory configuration

MIDClass is a Java application, and by default it starts with the default memory requirements established by most of the Java applications (usually 256 Megabytes). But most probably you will need more than the default, you will realize this in the case you obtain an exception like this:

java.lang.OutOfMemoryException
There is a way to configure the memory limits for MIDClass the same way that Java does, using the option -Xmx. But this configuration should be specified through an enviroment variable called **MIDCLASS_JAVA_OPTS**. Let's see some examples of how to do this with different operating systems:

Linux

Imagine that you would like to use 1024 megabytes of memory for MIDClass, then edit the **~/.bashrc** file adding a line like this:

export MIDCLASS_JAVA_OPTS='-Xmx1024m'
You can also specify 2 gigabytes like this:
export MIDCLASS_JAVA_OPTS='-Xmx2g'

Starting MIDClass

Open a Terminal window, cd into the MIDClass directory, and run the command: java -jar MIDClass.jar. When running on Linux/Unix OS, make sure that you have rwx permissions for the MIDClass directory and for the directory in which your data is located. Upon running the program, the GUI appears:

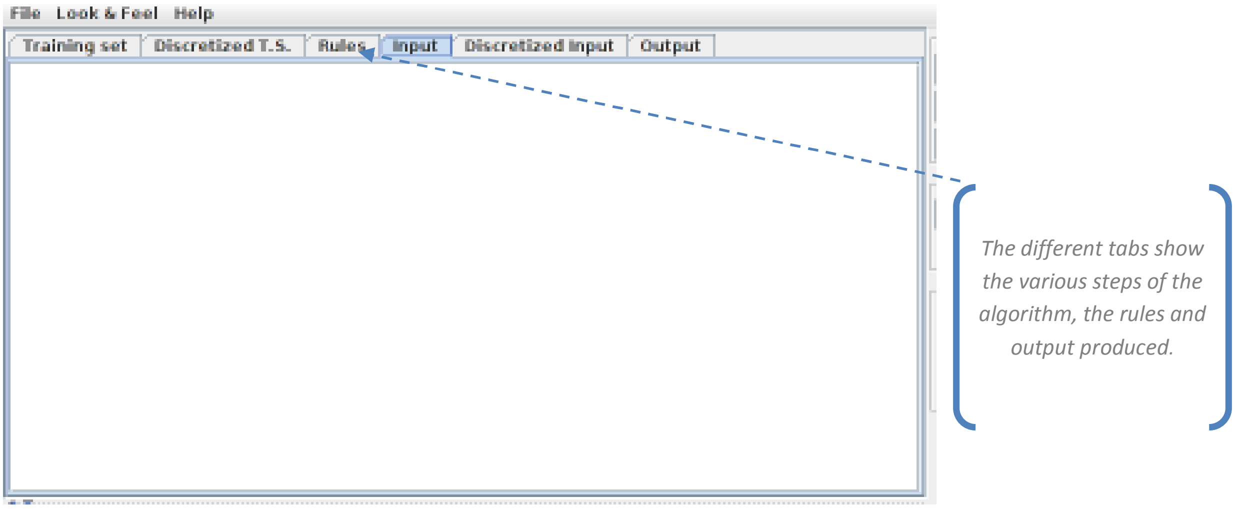

Description of Tabs

- Training Set: The tab shows the data matrix chose.
- Discretized T.S.: It shows the discretized matrix. For each gene, MIDClass discretizes the gene expression building this new matrix.
- Rules: It allows visualizing the discriminant association rules created by the algorithm.
- Input: The user can visualize the elements to be classified.
- Discretized Input: It shows the discretized input matrix.
- Output: In this tab, the program prints all input produced.

MIDClass setting and go fuction

The action buttons on the left of GUI perform all function provided by MIDClass; they are in the following paragraphs. Our algorithm has several parameters that users can set with the buttons present below the action buttons. However, we experimentally tuned the method to establish default values, with those values MIDClass outperformed all the other methods in almost all cases. Users can set:

- Discretization Function: Chose among ID3, CACC, USD, EWIB, EFIB, KMEANS, MC, ENTR, RMDL and SSD (default ID3).
- MFI Threshold: Cutoff for the maximal frequent itemsets (default 0.05),
- Minimum Interval size threshold: Allows to remove gene-intervals whose range is smaller than it (default 0.05).

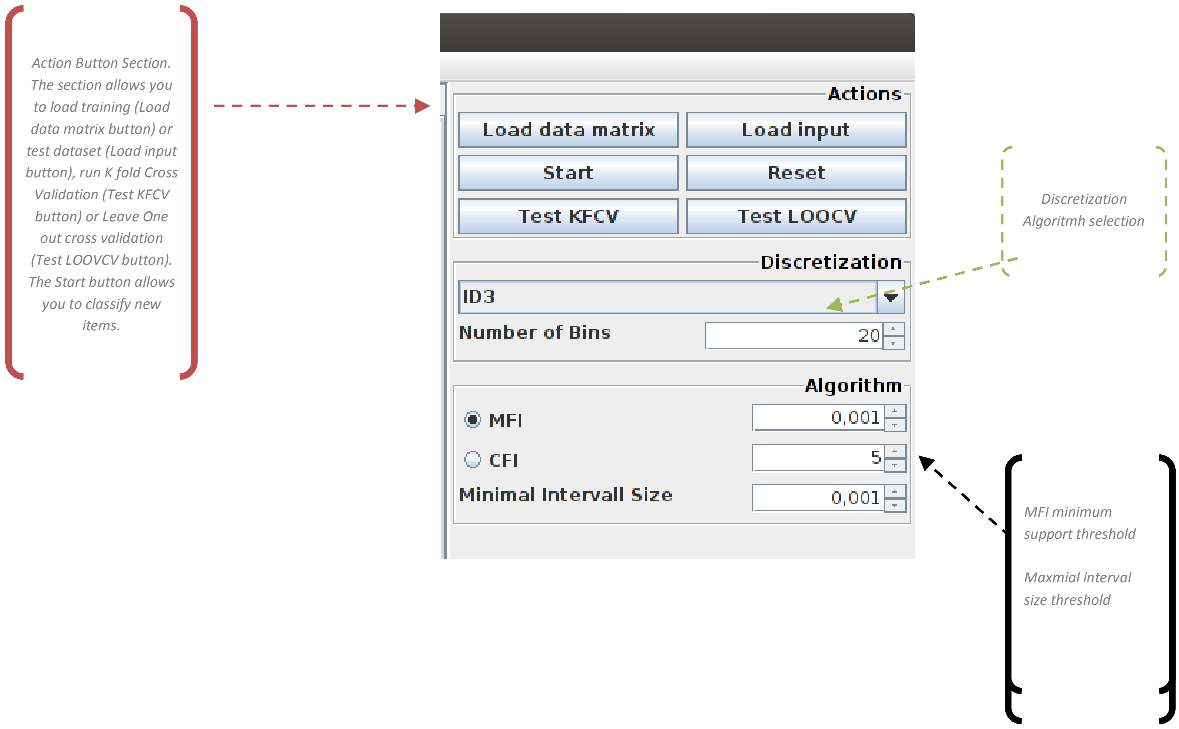

Input Data

The input data files should be tab-delimited ASCII text files. Each row in this file represents a gene/microRNA in the dataset (except for the first two rows which are a header row and the class row), and each column represents a sample. The header row must contains for each sample a unique label as alphanumeric string, the class row must contains for each sample the belonging class as alphanumeric label. Here is an example:

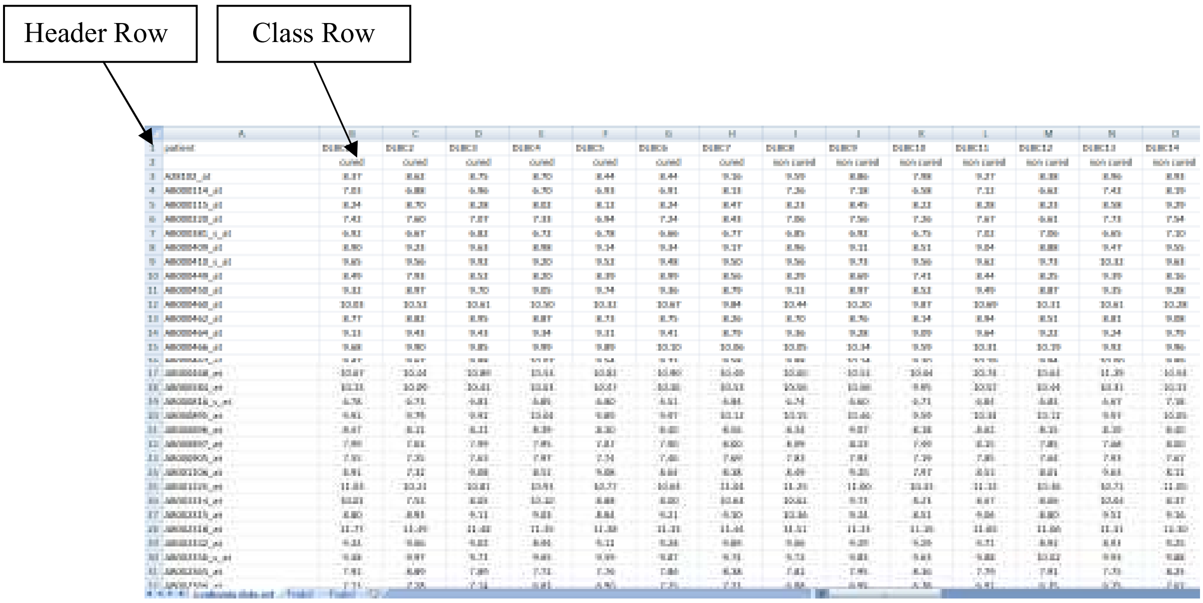

To load tabular expression data, select: *File >> load data Matrix* or click the button labelled “Input data matrix”. The dialog box will appear allowing you to select your input file.

Leave One Out and K-Fold Cross Validation

The buttons labeled with "Test LOOCV" and "Test KFCV" provide an estimate of how accurately the classes can be predicted by MIDClass method. The cross-validated misclassification rate is computed and the results are reported. The loocv process omits one sample at a time and in k-fold cross-validation, the original sample is randomly partitioned into k equal size subsamples. For each cross-validation step, the entire analysis is repeated from scratch: a predictor is constructed and applied to the sample or subsample that was omitted. The program records whether that prediction was correct or not. This is repeated, omitting all of the samples one at a time. The output table shows which samples were correctly and incorrectly predicted, and the overall cross-validated misclassification rate. The output is shown on "Output" tab and is arranged so that you can see the extent to which the different predictors agree or disagree for each specimen left out of the training set.

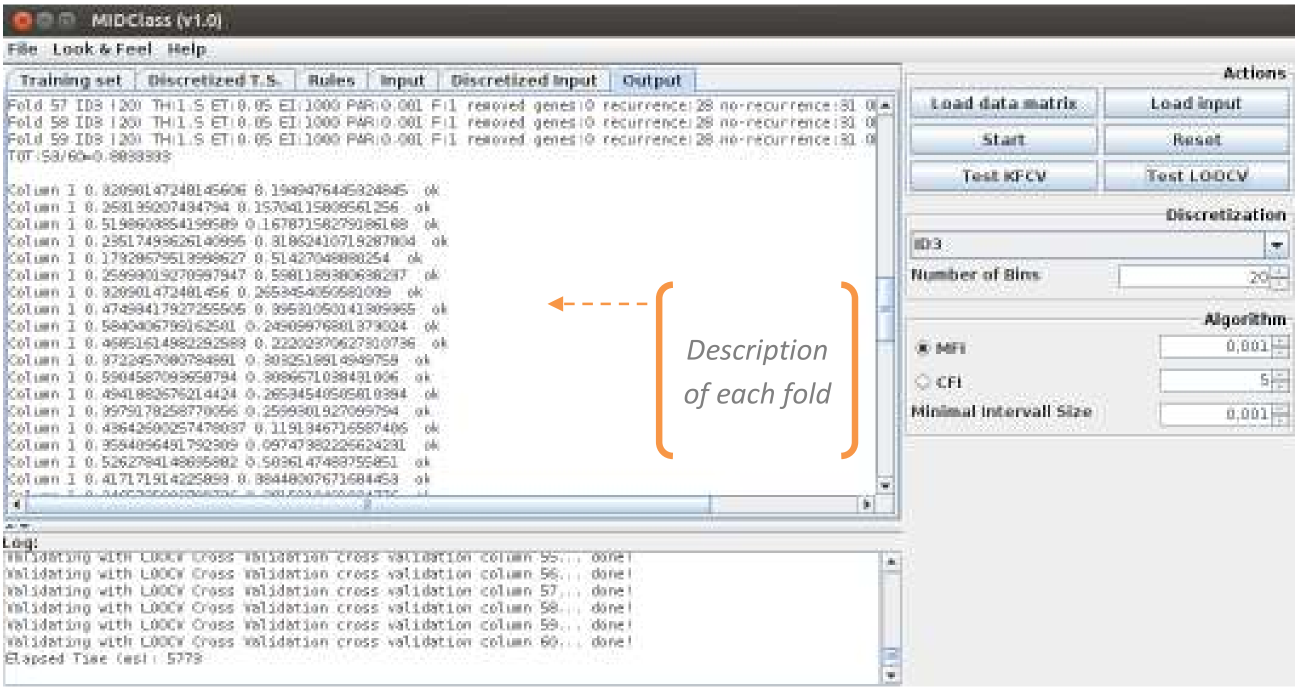

Prediction for new samples

Usually, you do not have a separate set of samples that you wish to withhold from the model building process when the analysis is first done. Sometimes, however, after the model is built, you have additional samples whose classes you wish to predict using the previously developed model. We have enabled you to do this by re-building the model using the initial set of samples, and to then predict for the new samples in a combined analysis.

To classify samples not used in the model building process. The input data files should be tab-delimited ASCII text files. Each row in this file represents a gene/microRNA in the dataset (except for the first row which is a header row) and each column represents a sample. To load tabular expression data, select: File >> Load Input or click the button labeled "Load Input". The dialog box will appear allowing you to select your input file.

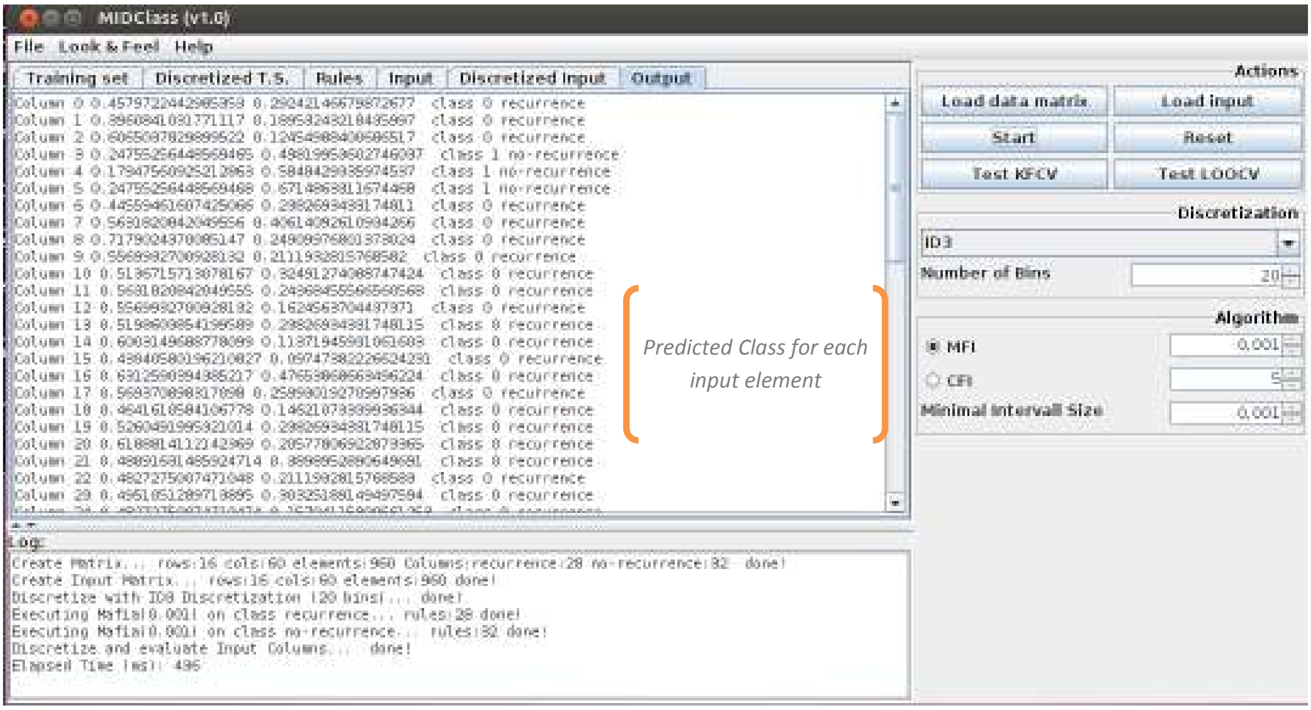

Then click the button labeled "Start" and you will find the results in the "Output" tab.
